# Boosting Delirium Identification Accuracy With Sentiment-Based Natural Language Processing: Mixed Methods Study

**DOI:** 10.2196/38161

**Published:** 2022-12-20

**Authors:** Lu Wang, Yilun Zhang, Mark Chignell, Baizun Shan, Kathleen A Sheehan, Fahad Razak, Amol Verma

**Affiliations:** 1 Department of Mechanical & Industrial Engineering University of Toronto Toronto, ON Canada; 2 Department of Computer Science Texas State University San Marcos, TX United States; 3 GEMINI - The General Medicine Inpatient Initiative Unity Health Toronto Toronto, ON Canada; 4 Department of Psychiatry University of Toronto Toronto, ON Canada; 5 Faculty of Medicine & Institute of Health Policy, Management and Evaluation University of Toronto Toronto, ON Canada

**Keywords:** delirium diagnosis, data mining, medical image description, text mining and analysis, sentiment analysis

## Abstract

**Background:**

Delirium is an acute neurocognitive disorder that affects up to half of older hospitalized medical patients and can lead to dementia, longer hospital stays, increased health costs, and death. Although delirium can be prevented and treated, it is difficult to identify and predict.

**Objective:**

This study aimed to improve machine learning models that retrospectively identify the presence of delirium during hospital stays (eg, to measure the effectiveness of delirium prevention interventions) by using the natural language processing (NLP) technique of sentiment analysis (in this case a feature that identifies sentiment toward, or away from, a delirium diagnosis).

**Methods:**

Using data from the General Medicine Inpatient Initiative, a Canadian hospital data and analytics network, a detailed manual review of medical records was conducted from nearly 4000 admissions at 6 Toronto area hospitals. Furthermore, 25.74% (994/3862) of the eligible hospital admissions were labeled as having delirium. Using the data set collected from this study, we developed machine learning models with, and without, the benefit of NLP methods applied to diagnostic imaging reports, and we asked the question “can NLP improve machine learning identification of delirium?”

**Results:**

Among the eligible 3862 hospital admissions, 994 (25.74%) admissions were labeled as having delirium. Identification and calibration of the models were satisfactory. The accuracy and area under the receiver operating characteristic curve of the main model with NLP in the independent testing data set were 0.807 and 0.930, respectively. The accuracy and area under the receiver operating characteristic curve of the main model without NLP in the independent testing data set were 0.811 and 0.869, respectively. Model performance was also found to be stable over the 5-year period used in the experiment, with identification for a likely future holdout test set being no worse than identification for retrospective holdout test sets.

**Conclusions:**

Our machine learning model that included NLP (ie, sentiment analysis in medical image description text mining) produced valid identification of delirium with the sentiment analysis, providing significant additional benefit over the model without NLP.

## Introduction

### Background

Delirium is described as “acute brain failure” and is considered both a “medical emergency” and “quiet epidemic” [1,2]. It is the most common neuropsychiatric condition among medically ill and hospitalized patients [3]. It is also recognized as a quality of care indicator in Canada, the United States, the United Kingdom, and Australia [4-8]. Symptoms of delirium can be severe and distressing for both patients and caregivers [9,10] and result from a complex interaction between predisposing and precipitating factors [9]. Affecting up to 50% of older hospital patients, those with delirium are more than twice as likely to die in the hospital or require nursing home placement [11-14]. The long-term effects of delirium are serious, as it is associated with worsening cognitive impairment and incident dementia [14-17]. Patients with delirium have longer hospitalizations, increased readmission rates, and more than double the health care costs. The study by Leslie et al [18] indicated that 1-year health costs associated with delirium ranged from US $16,303 to US $64,421 per patient. More recent estimates suggest that it accounts for US $183 billion dollars of annual health care expenditures in the United States [18,19]. Up to 40% of cases are preventable and many of the remaining cases of delirium could be better managed with implementation of standardized multicomponent programs [19,20]. These programs result in up to US $3800 in savings per patient in hospital costs and >US $16,000 in savings per person-year in the year following an episode of delirium [19,20]. However, in routine clinical care, there is a significant practice gap, and most hospitals have not consistently implemented best practices [19-21].

A key barrier in using delirium as a quality indicator is the lack of a reliable and scalable method for early identification of delirium cases. Clinicians are not good at recognizing delirium using clinical gestalt, with corresponding recognition rates ranging between 16% and 35% [22]. The Confusion Assessment Method (CAM) [23] is one of the number of screening tools for delirium, but it takes time and training to use; as a result, tools such as CAM are used relatively infrequently. For instance, Hogan et al [23] found that only 28% of emergency departments with a geriatric focus used delirium screening tools.

As delirium is difficult to recognize in situ, there has been interest in recognizing delirium after it has occurred, either through administrative chart review (ie, looking for evidentiary factors such as the use of antipsychotic drugs) or through retrospective identification. Ideally, identification of delirium would be prospective, proving a method to identify those at the highest risk of developing delirium to target delirium identification interventions for these individuals. However, retrospective identification of delirium can also be useful in determining delirium rates, which can serve as quality indicators and measures of effectiveness for interventions aimed at quality improvement.

Numerous models for predicting delirium have been developed based on known predisposing and precipitating risk factors [18]. However, current models have limitations [24]. First, they rely on variables not routinely collected as part of clinical care such as preexisting cognitive impairment and functional status, making them difficult to scale [25]. For example, the United Kingdom’s National Institute for Clinical Excellence delirium risk identification model requires information on cognitive impairment and sensory impairment to be available in the electronic record [26-28]. Second, a systematic review of delirium identification models highlighted their inadequate identification and numerous methodological concerns regarding how the models were validated such as their accuracy and inadequate predictive ability. The review concluded that model performance was likely exaggerated [26]. Third, prior risk identification models for delirium have tended to use a limited set of machine learning methods [7,29-33] and have tended to neglect text data [34].

With the growing availability of electronic clinical data repositories such as the one used in this study, methods such as data mining and machine learning can supplement or replace conventional statistical models [27,32,34-38]. Natural language processing (NLP) methods for medical text mining are required to extract valuable medical information and derive calculable variables for identification models [39]. NLP has proven to be highly effective in extracting the information from medical text into a computationally useful form that can support clinical decision-making [40-47].

Sentiment analysis analyzes the text for the sentiment of the writer (eg, positive vs negative, or in our case delirium vs non–delirium-related text) using machine learning and NLP [46-48]. We adapted sentiment analysis to predict sentiment concerning delirium status. Thus, positive (with delirium) and negative (without delirium) status was a new (binary) sentiment feature in the subsequent analysis. Using this delirium-based text sentiment analysis, we created a text-derived feature that estimated the delirium status for each admission.

### Objective

The overall research goal of our project was to retrospectively identify delirium cases during hospitalization using all data available from admission to discharge to estimate delirium rates and thereby quantify the effect of quality improvement interventions related to delirium. In this study, we focus on the methodological goal of demonstrating the value of incorporating NLP methods in the retrospective identification of delirium.

## Methods

### Data Source

#### Overview

The General Medicine Inpatient Initiative (GEMINI) is a multi-institutional research collaboration in Ontario, Canada. GEMINI has developed infrastructure and methods to collect and standardize electronic clinical data from hospitals. The data for this study were obtained from 6 hospitals (St Michael’s Hospital, Toronto General Hospital, Toronto Western Hospital, Trillium Credit Valley Hospital, Trillium Mississauga Hospital, and Sunnybrook Hospital). GEMINI is emerging as a rich resource for clinical research and quality measurement [4,49-52]. A rigorous internal validation process demonstrated 98% to 100% accuracy across key data types [50].

In GEMINI, administrative health data are linked to clinical data extracted from hospital information systems at the individual patient level (Table 1).

**Table 1:** Data contained in the General Medicine Inpatient Initiative project.

Data type	Patient details	Physician and room	Laboratory	Imaging	Pharmacy	Clinical documentation	Microbiology
Selected variables	Demographics Comorbidities Diagnoses Procedures Costs	Physician details Transfer details	Biochemistry Hematology Transfusion	Radiologist reports of diagnostic and interventional imaging	Medication Dose Route	Physician orders Vital signs	Organism Antimicrobial susceptibility Collection details

#### Administrative Data

Patient-level characteristics were collected from hospitals as reported to the Canadian Institute for Health Information Discharge Abstract Database and the National Ambulatory Care Reporting System. Diagnostic data and interventions were coded using the enhanced Canadian International Statistical Classification of Diseases and Related Health Problems and the Canadian Classification of Health Interventions.

#### Clinical Data

Data from the electronic information systems in GEMINI include laboratory test results (biochemistry, hematology, and microbiology), blood transfusions, in-hospital medications, vital signs, imaging reports, and room transfers. The quality of the key elements of these data was ensured through statistical quality control processes and direct data validation [53]. GEMINI data extraction methods allow access to a wealth of data ideal for text processing methods, including radiologist reports of diagnostic imaging.

The delirium cases in the research reported here were identified through manual medical record review by trained medical professionals using a validated method [54]. This method relies primarily on the identification of delirium or its numerous synonyms (eg, confusion) through a detailed review of physicians, nurses, and interprofessional documentation. The method has good sensitivity (74%) and specificity (83%) compared with clinical assessment and is considered a suitable gold standard for the identification of delirium for research and quality improvement [54].

We used 11 data files from a GEMINI data set that contained 3862 hospital admissions manually labeled according to delirium status. The data files include clinical and administrative data, as described in Table 1. However, labeling delirium is highly labor intensive, with trained reviewers answering the following question as part of the process: “Is there any evidence from the chart of acute confusional state (e.g., delirium, mental status change, inattention, disorientation, hallucinations, agitation, inappropriate behavior, etc.)? Review the entire medical record, including progress notes, nursing notes, consult notes, etc.” Thus, although chart review labels can be used to train more efficient machine learning methods, they are too expensive to use in label all older patients in terms of whether they experienced delirium during their hospital stay.

In our study, we used the chart review method [51] to label a subset of cases in our data set with respect to delirium. Interrater reliability was assessed by having 5% of the charts blindly reviewed by a second abstractor, achieving 90% interrater reliability. This resulted in the 3862 hospital admissions used in the analyses reported in this paper. The data files include clinical and administrative data, as described in Table 1.

### Ethics Approval

The research ethics board (REB) at the Toronto Academic Health Science Network approved the GEMINI study (REB reference number 15-087). The extension of the REB approval was issued by the Unity Health Toronto REB (reference number 15-087). A separate REB approval was obtained for Trillium Health Partners.

This paper is also part of the GEMINI substudy, named “Using artificial intelligence to identify and predict delirium among hospitalized medical patients,” which was approved by the University of Toronto REB (approved as reference number 38377).

### Data Preprocessing

The data tables contained in GEMINI were merged into a single table worksheet suitable for conducting machine learning. Before that, merger relevant variables were selected from the data tables, as described in the following subsections.

#### Laboratory Tests

A total of 45 medical tests were included in this data file, for example, blood urea nitrogen, mean cell volume, and high sensitivity troponin. Note that in each admission, not all 45 medical tests were performed, although some tests were performed several times in the same patient. In the original laboratory tests data file, each instance of a medical test corresponded to a separate record. We converted the laboratory tests table to one with a single row per admission, where each column represented a different test. As patients typically received a small subset of the available tests, there were many empty cells (ie, sparsity), and some cells had to represent multiple instances of the same test. To address the problem of sparse variables, we converted them to 1 or 0 flag variables (1 for test performed and 0 for test not performed). For frequently performed tests, we recorded the minimum, maximum, median, and frequency of the test results for each admission. If a test was administered at least five times in >50% of the admissions, we calculated the SD of the test results across each admission as an additional summary measure.

#### Patient Diagnosis

We first mapped the International Classification of Diseases, Tenth Revision (ICD-10) to the Clinical Classification Software (CCS) discharge diagnosis codes in a process that we previously described [4,49,50,55]. We use all available ICD-10 codes, including those assigned retrospectively, and this should not be considered data leakage but rather leveraging all available data to serve the use. The physician team identified 240 unique CCS codes potentially relevant to delirium. We then created flag variables (Boolean) for these 240 unique CCS codes that indicated whether the admission involved each of the diagnoses. Note that we did not create flag variables for ICD-10 codes because this would have dramatically increased the number of features in the analysis.

#### Clinical Interventions

This set of features covered a range of clinical interventions including surgical and endoscopic procedures as coded by the Canadian Classification of Health Interventions. Two variables were used to record the number of interventions for each admission. The first derived variable was the number of interventions performed per admission (including repetitions of the same intervention). The second derived variable counted the number of unique interventions per admission. No other information regarding interventions was used in the data file.

#### Room Transfers

We calculated the number of room transfers for each admission, which was the only variable used from this data table.

#### Clinical Risk Scores

We used the following clinical scores, which are markers of illness severity and patient risk of adverse outcomes: Charlson Comorbidity Index [56], Laboratory-Based Acute Physiology Score [57], and Kidney Disease: Improving Global Outcomes Acute Kidney Injury stages [58].

#### Emergency Department Triage Score

We applied one-hot encoding on the feature representing the patient’s illness severity at the time of emergency department triage with a 5-point scale, as measured by the Canadian Triage and Acuity Scale [59].

#### Administrative Admission and Discharge Data

We applied one-hot encoding on the feature representing the type of medical services that the patient was admitted to and discharged from as per the hospital admission, discharge, and transfer system. We also calculated hospital length of stay and derived a feature to indicate where the patient was discharged to.

#### Medications

This file had 1 row per admission and was used as is.

#### Special Care Unit

Only 320 admissions had special care unit information, so we created a flag variable with binary coding to indicate whether patients were cared for in a special care unit at any point during the admission.

#### Blood Transfusion

This medical data table contained only 429 admissions that included blood transfusion information; therefore, we created 1 column with binary coding to represent its presence or absence.

### NLP on Radiologist Reports of Diagnostic Imaging

The medical imaging data table contained the text description of magnetic resonance images and computed tomography scans, which were filtered to include only brain or head imaging. Similar to the laboratory tests data file, there was 1 row per imaging test; therefore, there could be multiple rows per admission. If there were multiple tests per admission, we first concatenated the text descriptions across the tests and then used text mining on this file by cleaning, tokenizing, and vectorizing.

The data set used for machine learning represented data integrated from multiple sources, for example, laboratory results, medications, radiologist reports, and administrative data. We adapted sentiment analysis to predict sentiment concerning delirium status. Thus positive (with delirium) and negative (without delirium) status was a binary sentiment that then formed a new feature in the subsequent analysis. Using this delirium-based text sentiment analysis, we created a text-derived feature that estimated the delirium status for each admission.

Preliminary text analysis was carried out before the sentiment analysis. Text cleaning included uppercase transformation, stop words removal, punctuation removal, intraword splitting, tokenization, and lemmatization and was performed using the *nltk* [39] and *sklearn* [60] packages. Next, term frequency–inverse document frequency, word count representation, and *n*-gram methods were applied for text vectorization.

A total of 8 baseline machine learning classification models were then trained to perform sentiment analysis, that is, logistic regression, Naive Bayes, support vector machine (SVM), decision tree, random forest, gradient boosting, *XGboost*, and multilayer perceptron. Hyperparameter tuning was applied using *RandomSearchCV* (ie, a randomized search on hyperparameters optimized by cross-validated search over parameter settings) [60].

Gradient boosting was selected as the final sentiment analysis method because its *F*_1_-score was the highest among the 8 classifiers. The final model was a stochastic gradient boosting (with a 0.8 subsample) that used 200 estimators, with Friedman mean square error as the criterion and a maximum depth of 3. We then created a feature with the predicted binary sentiment from the description of the medical images in the text using the selected gradient boosting model.

We integrated this new feature with 10 laboratory tests and electronic health record data to create a complete data file for training and testing machine learning identification models.

### Model Construction and Training

A total of 12 supervised classification algorithms with the task of predicting delirium status were implemented. The 12 machine learning algorithms covering most types of machine learning models were as follows:

- Ensemble machine learning models: gradient boosting classifier, AdaBoost classifier, random forest, and voting classifier soft
- Nonparametric machine learning models: K-nearest neighbor and decision tree
- Linear parametric machine learning models: logistic regression, linear SVM, and linear discriminant analysis
- Nonlinear parametric machine learning models: quadratic discriminant analysis, neural network: multilayer perceptron classifier in deep learning
- Bayesian-based machine learning models: Gaussian Naive Bayes.

**Figure 1:**
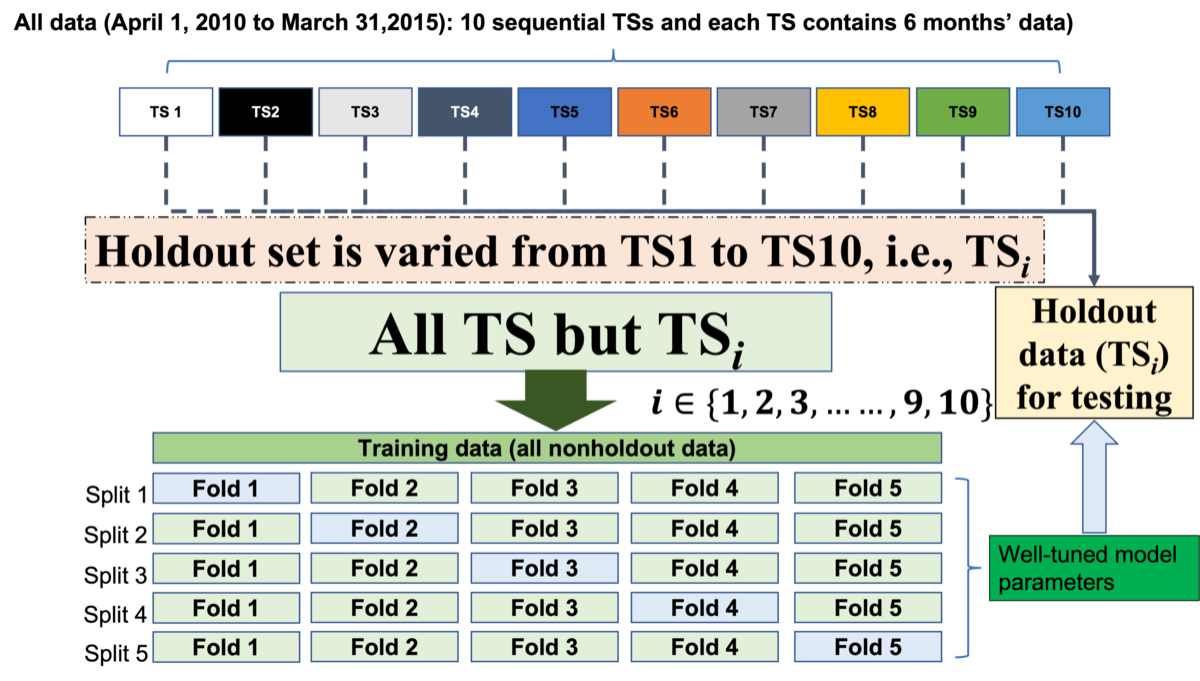
Data splits for models training and testing on a rolling basis. TS: time segment.

For the modeling, we split our integrated complete data into 2 parts, a training set and a testing set. As shown in Figure 1, the data extended over a 5-year period, from April 1, 2010, to March 31, 2015. We divided this period into ten 6-month segments. We treated the first 9 segments, that is, April 1, 2010, to September 30, 2014, as the training set. The last 6-month period, that is, October 1, 2014, to March 1, 2015, was used as holdout data (ie, testing set) to estimate the likely future performance of the model that was forward in time relative to the data used in building the model. This allowed us to assess whether there was any nonstationarity in the data, which would affect our ability to predict delirium in the future based on models developed on currently available data as transferability to future data.

In the training set, we used 5-fold cross-validation to tune the model parameters for each of the 12 machine learning algorithms. We then used the tuned parameters from the 5-fold cross-validation to identify delirium status of each admission in the testing or holdout set.

## Results

### Overview

We tested the model performance on the holdout testing set and calculated 6 evaluation metrics to find the best model, that is, accuracy, precision, recall or sensitivity, *F*_1_-score, specificity, and area under the receiver operating characteristic curve (ROC-AUC).

Accuracy answers the question of how many admissions did we correctly label out of all the admissions.

Precision answers the question of how many of those who we predicted as having delirium actually had delirium.

Sensitivity represents the proportion of people with delirium who were correctly labeled as having delirium.

*F*_1_-score is a weighted average of the precision or recall, where the *F*_1_-score reaches its best value at 1 and worst score at 0.



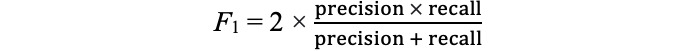



Specificity answers the question of how many negative instances (ie, people with no delirium) were correctly predicted.

The ROC curve was plotted using the true-positive rate against the false-positive rate at various threshold settings. The calculated ROC-AUC indicated the probability that our binary classifier ranked a randomly chosen positive instance higher than a randomly chosen negative one (assuming “positive” ranks higher than “negative”).

The 12 machine learning algorithms, along with hyperparameter tuning and cross-validation, were implemented in the Python package *Scikit-learn* [60]. Hyperparameter tuning was conducted using the *RandomizedSearchCV* and *GridSearchCV* functions. Cross-validation was used via the *cross_val_score*, *cross_validate,* and *cross_val_predict* functions.

The gradient boosting classifier was trained using the *GradientBoostingClassifier* function. The AdaBoost classifier used the *AdaBoostClassifier* function. The neural network classifier was implemented using the *MLPClassifier* function. The decision tree classifier was implemented using the *DecisionTreeClassifier* function. K-nearest neighbor classification was trained using the *KNeighborsClassifier* function. The logistic regression classifier used the *LogisticRegression* function. The random forest classifier was implemented using the *RandomForest* classifier function. The SVM method used the *svm* function. The Gaussian Naive Bayes method implemented the *GaussianNB* function. The linear discriminant analysis classifier was trained using the *LinearDiscriminantAnalysis* function. The quadratic discriminant analysis classifier used the *QuadraticDiscriminantAnalysis* function. The voting classifiers with soft settings were implemented using the *Voting Classifier* function.

### Experimental Results

We trained these models using hyperparameter tuning and 5-fold cross-validation on the first 9 time segments. We present the results from the 3 best-performing models in Table 2, and the results from the other 9 models are presented in Multimedia Appendix 1. In both tables, we report the average performance over 5 folds for the data from the first 9 time segments.

We then tested our delirium identification (sentimental or +NLP) model, which incorporated NLP in the training process. We compared the results of the +NLP model with the results obtained for the unsentimental (–NLP) delirium identification model that was trained, without NLP, on the last 6-month data in the GEMINI data set. The performance of the 3 best-performing models in predicting delirium labels in the last 6 months of the data is shown in Table 3. A similar presentation of the results is shown for the other 9 models in Multimedia Appendix 2. It should be noted that we used well-tuned parameters from the best-performing models of the training data on the testing data.

**Table 2:** Comparison of models in the 3 best-performing algorithms: average training results using 5-fold cross-validation on training set (April 1, 2010, to September 30, 2014).

Models		Gradient boosting classifier	AdaBoost classifier	Random forest
**Accuracy**				
	Delirium (+NLP^a^)	*0.868* ^b^	0.866	0.826
	Delirium (–NLP)	0.797	0.795	0.768
**Precision**				
	Delirium (+NLP)	0.78	0.794	*0.833*
	Delirium (–NLP)	0.747	0.75	0.8
**Recall**				
	Delirium (+NLP)	*0.678*	0.649	0.398
	Delirium (–NLP)	0.341	0.329	0.141
**Specificity**				
	Delirium (+NLP)	0.935	0.942	0.975
	Delirium (–NLP)	0.957	0.958	*0.988*
**ROC-AUC^c^**				
	Delirium (+NLP)	*0.91*	0.895	0.897
	Delirium (–NLP)	0.83	0.834	0.83
***F*_1_-score**				
	Delirium (+NLP)	*0.722*	0.712	0.529
	Delirium (–NLP)	0.463	0.452	0.239

^a^NLP: natural language processing.

^b^Highest performance values are italicized.

^c^ROC-AUC: area under the receiver operating characteristic curve.

**Table 3:** Comparison of 3 types of models in the 3 best-performing algorithms: model performance on holdout set 10 (October 1, 2014, to March 31, 2015).

Models		Gradient boosting classifier	AdaBoost classifier	Random forest
**Accuracy**				
	Delirium (+NLP^a^)	*0.853* ^b^	0.835	0.835
	Delirium (–NLP)	0.807	0.811	0.776
**Precision**				
	Delirium (+NLP)	0.742	0.725	*0.866*
	Delirium (–NLP)	0.74	0.747	0.806
**Recall**				
	Delirium (+NLP)	*0.669*	0.594	0.436
	Delirium (–NLP)	0.406	0.421	0.188
**Specificity**				
	Delirium (+NLP)	0.918	0.92	0.976
	Delirium (–NLP)	0.949	0.949	*0.984*
**ROC-AUC^c^**				
	Delirium (+NLP)	0.922	0.917	*0.93*
	Delirium (–NLP)	0.848	0.849	0.869
***F*_1_-score**				
	Delirium (+NLP)	*0.704*	0.653	0.58
	Delirium (–NLP)	0.524	0.538	0.305

^a^NLP: natural language processing.

^b^Highest performance values are italicized.

^c^ROC-AUC: area under the receiver operating characteristic curve.

In the training set, our proposed delirium (+NLP) models performed the best in terms of accuracy, precision, recall or sensitivity, rate, ROC-AUC, and *F*_1_-score, whereas delirium (–NLP) models generated the best specificity. In the testing set, the performances in both delirium (+NLP) and delirium (–NLP) models continued the same trend.

Note that *F*_1_-score is the balance of sensitivity and precision, and ROC-AUC is generated by sensitivity and specificity so that our delirium (+NLP) models performed the best in terms of balancing sensitivity, precision, and specificity. In acute diseases such as delirium, sensitivity is particularly important because the cost of failed identification of a disease (a miss) is higher than the cost of a false alarm. Thus, the present results indicate that the sentimental (vs unsentimental) delirium identification model should be more useful in clinical practice.

We also tested the +NLP and –NLP models across time, moving the holdout set across each of the 9 time segments one at a time, before using the most recent time segment as the holdout set. Thus, each of the time segments was used as the testing set, whereas the other 9 time segments were treated as the training set on a rolling basis, as shown in Figure 1. The corresponding data distribution of training and independent holdout or testing data are presented in Table 4. Tables 5 and 6 present the data distribution of patient characteristics of the cohort across the data splits.

**Figure 2:**
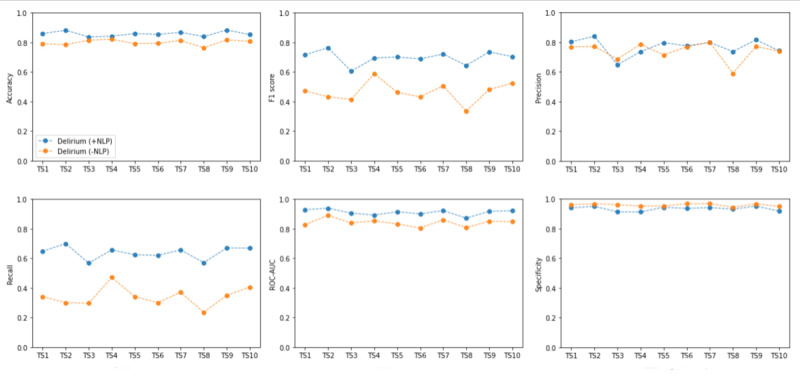
The performances of 2 schemes changing over the 10 time segments (TSs) are shown using the gradient boosting classifier, where TS1 to TS10 are as follows: April 1, 2010, to September 30, 2010; October 1, 2010, to March 31, 2011; April 1, 2011, to September 30, 2011; October 1, 2011, to March 31, 2012; April 1, 2012, to September 30, 2012; October 31, 2012, to March 31, 2013; April 1, 2013, to September 30, 2013; October 1, 2013, to March 31, 2014; April 1, 2014, to September 30, 2014; and October 1, 2014, to March 31, 2015. NLP: natural language processing; ROC-AUC: area under the receiver operating characteristic curve.

Figure 2 shows the identification results for the best-performing machine learning algorithm, that is, the gradient boosting across the 10 time segments. The 8 panels in the figure represent the 8 evaluation metrics used.

Note that the 2 different lines shown in each of the 8 panels within Figure 2 represent the results on the corresponding evaluation metrics for the 2 different types of models (ie, Delirium [+NLP] and Delirium [–NLP]). The 10 data points in each line show how the performance varied as the timing of the holdout time segment varied. Overall, the identification performance of the sentimental (+NLP) model was better than that of the unsentimental (–NLP) model. In addition, the performance of the sentimental (+NLP) model tended to be more stable across the different time segments than the other schemes. It can also be seen that precision, recall, and *F*_1_-score tended to be less stable over time than the other 3 measures, even though these performance measures remained relatively stable for the delirium (+NLP) model.

**Figure 3:**
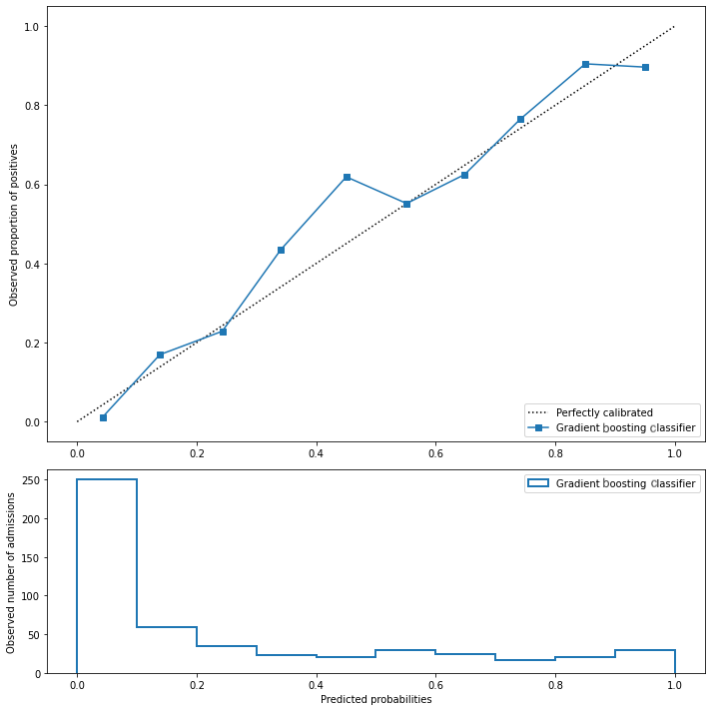
The calibration plot of the gradient boosting classifier.

Figure 3 presents the calibration of the gradient boosting model that was found to provide the best overall performance.

**Table 4:** Data distribution of training and holdout sets for each time segment (TS). Note that positive admissions indicate that the patients were diagnosed with delirium upon their admissions, whereas negative admissions were not.

Different TS as holdout set on a rolling basis	Training set				Holdout set		
	Number of admissions	Number of negative admissions	Number of positive admissions	Number of admissions		Number of negative admissions	Number of positive admissions
TS1	3541	2635	906	321		233	88
TS2	3531	2627	904	331		241	90
TS3	3494	2581	913	368		287	81
TS4	3488	2596	892	374		272	102
TS5	3526	2620	906	336		248	88
TS6	3479	2585	894	383		283	100
TS7	3446	2560	886	416		308	108
TS8	3476	2580	896	386		288	98
TS9	3424	2536	888	438		332	106
TS10	3353	2492	861	509		376	133

**Table 5:** Data information in patient characteristics for age and gender of the cohort across in 10 time segments (TSs) in both training and testing data sets. Three adult age groups are defined: young adults aged 18-44 years, middle-aged adults aged 45-64 years, and older adults aged ≥65 years.

TS	Gender				Age						
	Training		Testing		Training				Testing		
	Male, n (%)	Female, n (%)	Male, n (%)	Female, n (%)	Young adults, n (%)	Middle-aged adults, n (%)	Older adults, n (%)	Young adults, n (%)		Middle-aged adults, n (%)	Older adults, n (%)
TS1 (training: n=3541; testing: n=321)	1753 (49.51)	1788 (50.49)	162 (50.5)	159 (49.5)	430 (12.14)	844 (23.84)	2267 (64.02)	36 (11.2)		81 (25.2)	204 (63.5)
TS2 (training: n=3531; testing: n=331)	1736 (49.16)	1795 (50.84)	179 (54.1)	152 (45.9)	421 (11.92)	845 (23.93)	2265 (64.15)	45 (13.6)		80 (24.2)	206 (62.2)
TS3 (training: n=3494; testing: n=368)	1746 (49.97)	1748 (50.03)	169 (45.9)	199 (54.1)	417 (11.93)	845 (24.18)	2232 (63.88)	49 (13.3)		80 (21.7)	239 (64.9)
TS4 (training: n=3488; testing: n=374)	1737 (49.8)	1751 (50.2)	178 (47.6)	196 (52.4)	415 (11.9)	854 (24.48)	2219 (63.62)	51 (13.6)		71 (18.9)	252 (67.4)
TS5 (training: n=3526; testing: n=336)	1748 (49.57)	1778 (50.43)	167 (49.7)	169 (50.3)	423 (12)	838 (23.77)	2265 (64.24)	43 (12.8)		87 (25.9)	206 (61.3)
TS6 (training: n=3479; testing: n=383)	1728 (49.67)	1751 (50.33)	187 (48.8)	196 (51.2)	417 (11.99)	832 (23.91)	2230 (64.1)	49 (12.8)		93 (24.3)	241 (62.9)
TS7 (training: n=3446; testing: n=416)	1700 (49.33)	1746 (50.67)	215 (51.7)	201 (48.3)	415 (12.04)	833 (24.17)	2198 (63.78)	51 (12.3)		92 (22.1)	273 (65.6)
TS8 (training: n=3476; testing: n=386)	1724 (49.6)	1752 (50.4)	191 (49.5)	195 (50.5)	423 (12.17)	826 (23.76)	2227 (64.07)	43 (11.14)		99 (25.65)	244 (63.21)
TS9 (training: n=3424; testing: n=428)	1702 (49.71)	1722 (50.29)	213 (48.6)	225 (51.34)	409 (11.95)	817 (23.86)	2198 (64.19)	57 (13.01)		108 (24.66)	273 (62.33)
TS10 (training: n=3353; testing: n=509)	1661 (49.54)	1692 (50.46)	254 (49.9)	255 (50.1)	424 (12.65)	791 (23.59)	2138 (63.76)	42 (8.25)		134 (26.33)	333 (65.42)

**Table 6:** Data information in patient characteristics for special care unit (SCU) of the cohort across the data splits.

TS^a^	Training		Testing	
	In SCU file, n (%)	Not in SCU file, n (%)	In SCU file, n (%)	Not in SCU file, n (%)
TS1 (training: n=3541; testing: n=321)	291 (8.22)	3250 (91.78)	27 (8.4)	294 (91.6)
TS2 (training: n=3531; testing: n=331)	292 (8.27)	3239 (91.73)	26 (7.8)	305 (92.1)
TS3 (training: n=3494; testing: n=368)	289 (8.27)	3205 (91.73)	29 (7.9)	339 (92.1)
TS4 (training: n=3488; testing: n=374)	285 (8.17)	3203 (91.83)	33 (8.8)	341 (91.2)
TS5 (training: n=3526; testing: n=336)	290 (8.22)	3236 (91.78)	28 (8.3)	308 (91.7)
TS6 (training: n=3479; testing: n=383)	282 (8.11)	3197 (91.89)	36 (9.4)	347 (90.6)
TS7 (training: n=3446; testing: n=416)	286 (8.3)	3160 (91.7)	32 (7.7)	384 (92.3)
TS8 (training: n=3476; testing: n=386)	282 (8.11)	3194 (91.89)	36 (9.3)	350 (90.7)
TS9 (training: n=3424; testing: n=428)	282 (8.24)	3142 (91.76)	36 (8.2)	402 (91.8)
TS10 (training: n=3353; testing: n=509)	283 (8.44)	3070 (91.56)	35 (6.9)	474 (93.1)

^a^TS: time segment.

As with the results for the last 6-month time segment, the delirium (+NLP) model also performed best using data from each of the earlier 9 time segments as the holdout set. The delirium (+NLP) model outperformed the delirium (–NLP) model in terms of accuracy, precision, recall or sensitivity, miss rate, ROC-AUC, and *F*_1_-score.

## Discussion

### Principal Findings

Overall, machine learning models incorporating NLP either outperformed or were competitive with models that did not incorporate NLP for predicting the presence of delirium. Performance of the delirium (+NLP) model was relatively weaker on the specificity metric, but that metric was highly variable across the different holdout sets suggesting that it is a less reliable measure of performance in this application. As shown in the recall measure, the delirium (+NLP) model was better at detecting true positives, that is, identifying delirium for the admissions or patients who had ground truth delirium labels. The delirium (+NLP) model also performed best out of the 4 schemes in terms of having consistently high performance in terms of sensitivity, *F*_1_-score (balancing sensitivity and precision), and ROC-AUC.

Prior risk identification models for delirium have tended to use a limited set of machine learning methods [7,29-33] and have tended to neglect text data [34]. In addition, most machine learning identification models to identify delirium only evaluate via simple partition of data (randomly partitioned 80%/20% for training and validating the classification model, respectively) or cross-validation [30,32,33]. In contrast, we used independent holdout or testing data (cross-validation in training data and totally separate testing data over time segments on the rolling basis, as shown in Figure 1), providing more rigorous testing of the identification model.

Previous research has found that routine clinical screening, using tools such as CAM, underreports up to 75% of delirium cases compared with clinical assessments for research [61-64]. Although we were not able to directly compare our model’s performance with CAM results on the same patients, it is well documented in the literature that routine clinical use of CAM is unreliable for research or quality measurement, reinforcing the need for a model such as the one we developed in this study. Notably, the Montreal Cognitive Assessment is primarily used for the assessment of stable cognitive impairment and not for delirium.

The delirium (+NLP) model provided the best balance between recognizing cases of delirium, where they existed, and not mislabeling nondelirium cases as delirium. The baseline delirium scheme performed better when detecting true negatives. This is likely because our GEMINI data set was unbalanced, with 75% of admissions being nondelirium; thus, a poorly tuned model can achieve better accuracy by being biased toward predicting nondelirium.

One way of dealing with the trade-off between precision and recall is to use the *F*_1_-score, which is the harmonic mean (average) of the precision and sensitivity or recall scores. With this more balanced measure, our proposed delirium (+NLP) model outperformed the one without NLP across all time segments.

Our delirium (+NLP) method integrated an NLP derived feature into multisource medical data to improve the performance and usefulness of models. This approach can also be extended to other medical identification contexts.

This approach has several important applications, including for quality measurement and quality improvement, for statistical risk adjustment in research projects, and for large-scale observational research in retrospective cohorts. There is currently no scalable solution to retrospectively identify the occurrence of delirium in hospital, and CAM is underutilized, perhaps because of the lack of trained clinical resources. We agree that prospective predictions of delirium would be clinically useful, and research on that topic is underway. However, retrospective prediction is also important for quality management purposes and for evaluating the effectiveness of interventions for preventing delirium. Typically, CAM is poorly implemented and used infrequently [23].

One major reason why delirium is underidentified in routine data sources is because it is often inconsistently documented, with the use of various synonyms (eg, confusion and altered level of consciousness). The only validated, high-quality method for retrospectively identifying delirium is the Chart-based Delirium Identification Instrument review method that we used as the gold standard labeling method for training our machine learning models. This method is time intensive and requires up to 1 hour per hospital chart. Thus, it cannot be easily applied to large data sets. Therefore, developing models that can use routinely collected clinical and administrative health care data represents a major contribution to the literature, as they can enable both research and quality applications that rely on retrospective identification of delirium cases.

It would be desirable to build models that could predict delirium risk at the time of hospitalization or in real time during the course of hospital admission. One impediment to developing these models is having sufficiently large data sets on which to train them. Our models, which seek to accurately classify hospitalizations with or without delirium retrospectively could then be used to label (using model predictions) large data sets, which could then be used to generate quality estimates and provide a basis for further model prediction.

### Conclusions

Delirium is a highly prevalent, preventable, and treatable neurocognitive disorder, which is associated with very poor outcomes when untreated. It is characterized by an acute onset of fluctuating mental status, psychomotor disturbance, and hallucinations, and it is difficult to spot because the symptoms can often be attributed to other causes. Better delirium prediction will create an opportunity for higher quality care through automated identification of delirium or of delirium risk. In the research reported in this paper, we have shown that incorporation of the NLP approach can significantly improve identification compared with the standard machine learning methods without NLP. We also showed that varying the holdout period over time can estimate the temporal stability of model identification. Another useful feature of this type of stationarity analysis is that it can be used to identify unreliable evaluative criteria that exhibit nonstationarity and to identify models that are nonstationary with respect to their effectiveness over time. In this study, we found that precision was an unreliable criterion, with wide fluctuations over different periods.

The results of this study demonstrate the value of NLP in the identification of an important health care outcome, and we recommend that future research should focus on (1) applying NLP on medical notes to extract more valuable information and (2) augmenting the delirium (+NLP) model by adding explanations so that the resulting models are more consumable and more easily integrated into clinical workflow.

## Appendix

Multimedia Appendix 1 Comparison of models with average training results using 5-fold cross-validation on training set (April 1, 2010, to September 30, 2014) in the other 9 algorithms: neural network, decision tree, logistic regression, linear support vector machine, Gaussian Naive Bayes, linear discriminant analysis, quadratic discriminant analysis, and voting classifier.

Multimedia Appendix 2 Comparison of 3 types of models in the other 9 algorithms: model performance on holdout set 10 (October 1, 2014, to March 31, 2015).

## Abbreviations

CAM: Confusion Assessment Method
CCS: Clinical Classification Software
GEMINI: General Medicine Inpatient Initiative
ICD-10: International Classification of Diseases, Tenth Revision
NLP: natural language processing
REB: research ethics board
ROC-AUC: area under the receiver operating characteristic curve
SVM: support vector machine
