# Supplementary material for: Boosting Delirium Identification Accuracy With Sentiment-Based Natural Language Processing: Mixed Methods Study
Source: JMIR Med Inform. 2022 Dec 20;10(12):e38161. doi: 10.2196/38161 (PMC9812273; doi:10.2196/38161)
Supplement: Multimedia Appendix 1 [file medinform_v10i12e38161_app1.docx]

**Table 6.** Comparison of models with average training results using 5-fold cross validation on training set (2010.04.01-2014.09.30) in the other nine algorithms: neural network (NN), decision tree (DT), logistic regression (LR), linear SVM (SVM), Gaussian Naïve Bayes (GNB), linear discriminant analysis (LDA), quadratic discriminant analysis (QDA) and voting classifier (VC).

|  | **Models** | **NN** | **DT** | **kNN** | **LR** | **SVM** | **GNB** | **LDA** | **QDA** | **VC** |
| --- | --- | --- | --- | --- | --- | --- | --- | --- | --- | --- |
| **Accuracy** | Delirium (+NLP) | 0.765 | 0.854 | 0.746 | 0.86 | 0.668 | 0.73 | 0.854 | 0.418 | 0.778 |
|  | Delirium (-NLP) | 0.627 | 0.804 | 0.744 | 0.78 | 0.656 | 0.741 | 0.712 | 0.636 | 0.757 |
| **Precision** | Delirium (+NLP) | 0.619 | 0.766 | 0.547 | 0.768 | 0.354 | 0.476 | 0.75 | 0.293 | 0.611 |
|  | Delirium (-NLP) | 0.429 | 0.698 | 0.555 | 0.647 | 0.342 | nan | 0.442 | 0.389 | 0.701 |
| **Recall** | Delirium (+NLP) | 0.446 | 0.618 | 0.158 | 0.657 | 0.351 | 0.381 | 0.644 | 0.802 | 0.415 |
|  | Delirium (-NLP) | 0.809 | 0.447 | 0.087 | 0.367 | 0.368 | 0.0 | 0.418 | 0.687 | 0.108 |
| **Miss Rate** | Delirium (+NLP) | 0.554 | 0.382 | 0.842 | 0.343 | 0.649 | 0.619 | 0.356 | 0.198 | 0.585 5 |
|  | Delirium (-NLP) | 0.191 | 0.553 | 0.913 | 0.633 | 0.632 | 1.0 | 0.582 | 0.313 | 0.892 |
| **False Alarm** | Delirium (+NLP) | 0.124 | 0.063 | 0.048 | 0.07 | 0.221 | 0.148 | 0.073 | 0.716 | 0.095 |
|  | Delirium (-NLP) | 0.437 | 0.07 | 0.026 | 0.075 | 0.243 | 0.0 | 0.185 | 0.382 | 0.016 |
| **Specifivity** | Delirium (+NLP) | 0.876 | 0.937 | 0.952 | 0.93 | 0.779 | 0.852 | 0.927 | 0.284 | 0.905 |
|  | Delirium (-NLP) | 0.563 | 0.93 | 0.974 | 0.925 | 0.757 | 1.0 | 0.815 | 0.618 | 0.984 |
| **AOC-AUC** | Delirium (+NLP) | 0.793 | 0.846 | 0.667 | 0.896 | 0.518 | 0.715 | 0.898 | 0.546 | 0.863 |
|  | Delirium (-NLP) | 0.791 | 0.688 | 0.7 | 0.801 | 0.51 | 0.664 | 0.671 | 0.676 | 0.821 |
| **F1 Score** | Delirium (+NLP) | 0.474 | 0.675 | 0.239 | 0.704 | 0.35 | 0.412 | 0.692 | 0.411 | 0.481 |
|  | Delirium (-NLP) | 0.544 | 0.539 | 0.15 | 0.456 | 0.352 | 0.0 | 0.429 | 0.494 | 0.182 |
