# Supplementary material for: Boosting Delirium Identification Accuracy With Sentiment-Based Natural Language Processing: Mixed Methods Study
Source: JMIR Med Inform. 2022 Dec 20;10(12):e38161. doi: 10.2196/38161 (PMC9812273; doi:10.2196/38161)
Supplement: Multimedia Appendix 2 [file medinform_v10i12e38161_app2.docx]

**Table 7.** Comparison of three types of models in the other nine algorithms: model performance on holdout set 10 (2014.10.01-2015.03.31).

|  | | **Models** | | **NN** | **DT** | **kNN** | | **LR** | | **SVM** | | **GNB** | | **LDA** | | **QDA** | **VC** |
| --- | --- | --- | --- | --- | --- | --- | --- | --- | --- | --- | --- | --- | --- | --- | --- | --- | --- |
| **Accuracy** | Delirium (+NLP) | | 0.786 | | 0.792 | 0.782 | 0.807 | | 0.688 | | 0.731 | | 0.831 | | 0.393 | 0.778 | |
|  | Delirium (-NLP) | | 0.768 | | 0.809 | 0.745 | 0.802 | | 0.68 | | 0.739 | | 0.713 | | 0.66 | 0.76 | |
| **Precision** | Delirium (+NLP) | | 0.773 | | 0.639 | 0.806 | 0.667 | | 0.407 | | 0.477 | | 0.733 | | 0.295 | 0.635 | |
|  | Delirium (-NLP) | | 0.612 | | 0.68 | 0.556 | 0.716 | | 0.401 | | 0 | | 0.45 | | 0.415 | 0.789 | |
| **Recall** | Delirium (+NLP) | | 0.256 | | 0.466 | 0.218 | 0.526 | | 0.429 | | 0.308 | | 0.556 | | 0.955 | 0.353 | |
|  | Delirium (-NLP) | | 0.308 | | 0.511 | 0.113 | 0.398 | | 0.459 | | 0 | | 0.444 | | 0.737 | 0.113 | |
| **Miss Rate** | Delirium (+NLP) | | 0.744 | | 0.534 | 0.782 | 0.474 | | 0.571 | | 0.692 | | 0.444 | | 0.045 | 0.647 | |
|  | Delirium (-NLP) | | 0.692 | | 0.489 | 0.887 | 0.602 | | 0.541 | | 1 | | 0.556 | | 0.263 | 0.887 | |
| **False Alarm** | Delirium (+NLP) | | 0.027 | | 0.093 | 0.019 | 0.093 | | 0.221 | | 0.12 | | 0.072 | | 0.806 | 0.072 | |
|  | Delirium (-NLP) | | 0.069 | | 0.085 | 0.032 | 0.056 | | 0.242 | | 0 | | 0.191 | | 0.367 | 0.011 | |
| **Specifivity** | Delirium (+NLP) | | 0.973 | | 0.907 | 0.981 | 0.907 | | 0.779 | | 0.88 | | 0.928 | | 0.194 | 0.928 | |
|  | Delirium (-NLP) | | 0.931 | | 0.915 | 0.968 | 0.944 | | 0.758 | | 1 | | 0.809 | | 0.633 | 0.989 | |
| **AOC-AUC** | Delirium (+NLP) | | 0.742 | | 0.8 | 0.811 | 0.867 | | 0.411 | | 0.75 | | 0.886 | | 0.576 | 0.855 | |
|  | Delirium (-NLP) | | 0.808 | | 0.713 | 0.728 | 0.856 | | 0.417 | | 0.686 | | 0.688 | | 0.727 | 0.87 | |
| **F1 Score** | Delirium (+NLP) | | 0.384 | | 0.539 | 0.343 | 0.588 | | 0.418 | | 0.374 | | 0.632 | | 0.451 | 0.454 | |
|  | Delirium (-NLP) | | 0.41 | | 0.584 | 0.188 | 0.512 | | 0.428 | | 0 | | 0.447 | | 0.531 | 0.197 | |
